# Supplementary material for: Investigation of the Exposure of Schoolchildren to Ultrafine Particles (PM0.1) during the COVID-19 Pandemic in a Medium-Sized City in Indonesia
Source: Int J Environ Res Public Health. 2023 Feb 8;20(4):2947. doi: 10.3390/ijerph20042947 (PMC9957305; doi:10.3390/ijerph20042947)
Supplement: Supplementary file 1 [file ijerph-20-02947-s001.zip › ijerph-2113758-supplementary.pdf]

Supplementary Information (SI)

This supplementary information includes:
Equation (S1) to (S5)
Table S1 to S4

The respiratory deposition doses (RDDs) can be evaluated by:

$$RDD = DF_i \times (VT \times f) \times PM_i \quad \text{(S1)}$$

where *VT* is a tidal volume (m^3 breathe^{-1}), *f* is the typical breath frequency (breath min^{-1}), PM_i is the mass concentration of a size fraction *I*, and DF_{ij} is deposition fraction of a size fraction *i* and region *j*, e.g., head airways (DF_{HD}), tracheobronchial (DF_{TB}) and alveolar (DF_{AL}) regions.

The deposition fraction for the head airways region (DF_{HD}) was calculated as follows:

$$DF_{HD} = IF\left(\frac{1}{1+exp(6.84+1.183\ln d_p)}+\frac{1}{1+exp(0.924-1.885\ln d_p)}\right) \quad \text{(S2)}$$

where *dp* is the particle size in μm, while *IF* is the inhalable fraction calculated by:

$$IF = 1-0.5\left(1-\frac{1}{1+0.00076\, d_p^{2.8}}\right) \quad \text{(S3)}$$

The deposition fraction for the tracheobronchial region, DF_{TB}, was determined by:

$$DF_{TB} = \left(\frac{0.00352}{d_p}\right)\left[exp\left(-0.234(\ln d_p+3.40)^2\right)+63.9\, exp\left(-0.819(lnd_p-1.61)^2\right)\right] \quad \text{(S4)}$$

The deposition fraction for the alveolar region, DF_{AL}, was estimated by:

$$DF_{AL} = \left(\frac{0.0155}{d_p}\right)\left[exp\left(-0.416(lnd_p+2.84)^2\right)+19.11\, exp\left(-0.482(\ln d_p-1.362)^2\right)\right] \quad \text{(S5)}$$

Table S1. Description of schoolchildren participants for personal exposure measurement.

Student's origin	sex		home position			house occupant (people)		cooking duration/day (hour)				Smoking's at home		way to school	
	**boy**	**girl**	**near roadside**	**small road**	**housing estate**	**3-4**	**5-8**	**<1**	**1-2**	**3-4**	**≥5**	**yes**	**no**	**car**	**motor-cycle**
Urban schools															
U1	3	2	-	2	3	3	2	-	1	2	2	4	1	3	2
U2	1	7	-	8	-	5	3	1	3	2	2	4	4	-	8
U3	2	4	2	4	-	3	3	1	3	2	-	4	2	-	6
Total	6	13	2	14	3	11	8	2	7	6	4	12	7	3	16
Suburban schools															
SU1	3	5	1	6	1	7	1	-	-	4	4	5	3	-	8
SU2	-	7	1	1	5	4	3	-	1	2	4	6	1	-	7
Total	3	12	2	7	6	11	4	0	1	6	8	11	4	0	15
TOTAL	9	25	4	21	9	22	12	2	8	12	12	23	11	3	31

Table S2. Types of statistical tests used for data analyses.

No	Types of statistical analysis		description
1	Parametric tests	ANOVA	Comparison of schoolchildren's exposure to $PM_{<0.1}$, $PM_{0.1-0.4}$, and $PM_{0.4-1.0}$ based on their school origin and between their area of living (urban and suburban).
		Post-hoc test	Comparison details for the most significantly different of schoolchildren's exposure to $PM_{<0.1}$ and $PM_{0.1-0.4}$ following ANOVA result test based on their school origin.
2	Non-parametric tests	Kruskal-Wallis test	Comparison of schoolchildren's exposure to $PM_{1.0-2.5}$ based on their school origin.
		Mann-Whitney test	Comparison of schoolchildren's exposure to $PM_{1.0-2.5}$ between participants in urban schools and participants in suburban schools.

Table S3. General Characteristics of schoolchildren from five observed schools.

Characteristics	U1		U2		U3		SU1		SU2		Total		
	boys	Girls	boys	Girls	boys	Girls	boys	girls	boys	girls	boys	girls	all
7th grade (12-13 years old)	9	15	23	35	14	34	26	45	12	23	**84<br>35.6%**	**152<br>64.4%**	**236<br>32.8%**
8th grade (13-14 years old)	20	39	25	55	77	80	26	48	15	25	**163<br>39.8%**	**247<br>60.2%**	**410<br>57.0%**
9th grade (14-15 years old)	-	5	9	9	15	18	4	7	1	5	**29<br>39.7%**	**44<br>60.3%**	**73<br>10.2%**
	29<br>88 (12.2%)	59	57<br>156 (21.7%)	99	106<br>238 (33.1%)	132	56<br>156 (21.7%)	100	28<br>81 (11.3%)	53	**276<br>38.4%**	**443<br>61.6%**	**719 children**
**LIVING CONDITION**<br>**House location**													
near roadside	11	20	3	13	11	13	9	17	4	4	**38<br>36.2%**	**67<br>63.8%**	**105<br>14.6%**
small road	11	26	51	79	61	81	31	60	11	13	**165<br>43.2%**	**259<br>67.8%**	**424<br>59.0%**
housing estate	7	13	3	7	34	38	16	23	13	36	**73<br>38.4%**	**117<br>61.6%**	**190<br>26.4%**
**Family members**													
3 - 5 ORANG	21	40	40	67	76	93	46	64	24	36	**207<br>40.8%**	**300<br>59.2%**	**507<br>70.5%**
5 - 8 ORANG	6	16	14	29	23	35	10	31	4	17	**57<br>30.8%**	**128<br>69.2%**	**185<br>25.7%**
> 8 ORANG	2	3	3	3	7	4	-	5	-	-	**12<br>44.4%**	**15<br>55.6%**	**27<br>3.8%**
**Smoking status**													
Yes	15	38	39	68	58	56	38	63	20	38	**170<br>39.3%**	**263<br>60.7%**	**433<br>60.2%**
No	14	21	18	31	48	50	18	37	8	15	**106<br>40.8%**	**154<br>59.2%**	**260<br>36.2%**
**Cooking hours/day**													
2-3 hours	25	48	50	89	101	121	54	97	27	49	**257<br>38.9%**	**404<br>61.1%**	**661<br>91.9%**
4-5 hours	4	10	4	10	3	10	2	2	1	4	**14<br>28.0%**	**36<br>72.0%**	**50<br>7.0%**
more than 5 hours	-	1	3	-	2	1	-	1	-	-	**5<br>62.5%**	**3<br>37.5%**	**8<br>1.1%**
**TIME ACTIVITY DATA**<br>**Ways to school**													
walking	1	1	10	5	10	5	4	7	-	2	**25<br>5.7%**	**20<br>4.5%**	**45<br>6.3%**
motor vehicle	19	42	41	6	10	109	49	89	28	49	**147<br>33.3%**	**295<br>66.7%**	**442<br>61.5%**
car	8	15	4	84	82	11	2	4	-	2	**96<br>21.7%**	**116<br>26.2%**	**212<br>29.5%**
mass transportation	1	1	2	4	4	7	1	-	-	-	**8<br>1.8%**	**12<br>2.7%**	**20<br>2.8%**
**Schooling (hours)**	4	4	4	4	4	4	4	4	4	4	**16.7%**	**16.7%**	**70% at home,<br>16% in classrooms,<br>4% in commuting,<br>2.7% in outdoor.**
**Home-school transit (hours)**	<1	<1	<1	<1-1.5	<1-1.5	<1-1.5	<1	<1	<1-1.5	<1-1.5	**<4.2-6.3%**	**<4.2-6.3%**	
**At home-living room (hours)**	1-6	2-7	1-5	2-8	2-5.5	2-7	1-6	2-8	1-6	2-7	**4.2-25.0%**	**8.3-33.3%**	
**outside home (hours)**	<1-3	<1-2	1-2.5	<1-2	<1-3	<1-2	<1-3	1-2	1-3	<1-2	**<4.2-12.5%**	**<4.2-8.3%**	
**At home-sleeping (hours)**	6-9	6-9	6-9	6-9	6-9	6-9	6-9	6-9	6-9	6-9	**25.0-37.5%**	**25.0-37.5%**	
**HEALTH STATUS**<br>**Weight (kg) (average)**	51.3	45.2	46.9	44.0	49.3	46.0	40.1	41.0	49.7	39.5	**47.4**	**43.1**	**45.3**
**Height (cm) (average)**	158.5	156.2	154.8	154.3	158.2	156.1	144.1	153.7	154.4	142.3	**154.0**	**152.5**	**153.3**
**BMI for children (average)**	20.4	18.5	19.6	18.5	19.7	18.9	19.3	17.3	20.9	19.5	**20.0**	**18.5**	
**Respiratory problem**													
no	20	48	13	68	87	117	49	77	21	20	**190<br>38.9%**	**330<br>67.5%**	**520<br>72.3%**
common cold	3	3	`5	11	14	4	3	8	3	2	**23<br>45.1%**	**28<br>54.9%**	**51<br>7.1%**
cough	1	-	1	5	1	2	1	1	-	5	**4<br>23.5%**	**13<br>76.5%**	**17<br>2.4%**
cough and cold	1	6	2	4	3	3	3	5	1	-	**10<br>35.7%**	**18<br>64.3%**	**28<br>3.9%**
breathless	4	2	3	8	1	6	-	9	3	3	**11<br>28.2%**	**28<br>71.8%**	**39<br>5.4%**
**COVID-19 Symptoms**													
no	20	47	54	29	86	114	53	87	24	44	**237<br>42.5%**	**321<br>57.5%**	**558<br>77.6%**
ever	9	12	3	6	20	18	3	13	4	9	**39<br>73.6%**	**58<br>109.4%**	**97<br>13.5%**

Table S4. Time activity and microenvironments of schoolchildren participants during personal exposure sampling.

School	Student	at home (hours)				Total	transit (hours)		Total	School environments		Total	Others		Total
		Living room	Kitchen/dinner room	Bedroom	outside home		for schooling	for others		classroom	outside classroom		indoor	outdoor	
U1	1	2	1.75	1.25	1	**6**	0.5	0	**0.5**	3	1.5	**4.5**	0	0	**0**
	2	1.75	4	0.75	0	**6.5**	0.5	0	**0.5**	2.25	2.75	**5**	0	0	**0**
	3	2.5	1.75	1	1.25	**6.5**	0.5	0	**0.5**	2.75	1.75	**4.5**	0	0	**0**
	4	2.25	2.25	2	0	**6.5**	0.5	0.5	**1**	1.75	2.25	**4**	0	0	**0**
	5	2.25	0.5	2.75	2	**7.5**	0.75	0	**0.75**	2	1.75	**3.75**	0	0	**0**
U2	6	3.25	1.5	1.25	0.5	**6.5**	1	0	**1**	2.5	2	**4.5**	0	0	**0**
	7	1	2	1.25	0	**4.25**	0.5	0	**0.5**	3	0	**3**	0	3	**3**
	8	0.5	3	1.5	1	**6**	0.25	0	**0.25**	5	0.5	**5.5**	0	0	**0**
	9	0.75	3	3.25	0	**7**	0.5	0	**0.5**	2.75	0.75	**3.5**	0	0	**0**
	10	0.75	4.5	1	0.25	**6.5**	0.25	0.25	**0.5**	4	0.25	**4.25**	0	0	**0**
	11	1.5	2.5	2	0	**6**	0.5	0	**0.5**	2.75	1.25	**4**	0	0	**0**
	12	2	2	2	0	**6**	0.25	0	**0.25**	1.25	1.75	**3**	1	0	**1**
	13	1	3	2	0	**6**	0.25	0	**0.25**	2.25	0.75	**3**	1	0	**1**
U3	14	1.75	1.25	2	1	**6**	0.5	0.5	**1**	2	3	**5**	0	0	**0**
	15	0.25	4	1	0	**5.25**	0.25	0	**0.25**	3	0.5	**3.5**	0	1	**1**
	16	4.75	0.25	0	0	**5**	0.5	0	**0.5**	2.75	0.25	**3**	0	2	**2**
	17	2.75	1.25	2.5	0	**6.5**	0.25	0.5	**0.75**	1.75	1.75	**3.5**	0	0	**0**
	18	1.25	2.5	2.25	1	**7**	0.25	0.75	**1**	4	0	**4**	0	0	**0**
	19	1.5	0.5	3	0	**5**	0.25	0.25	**0.5**	3	0	**3**	2	0	**2**
SU1	20	0.75	1.5	2.5	1.25	**6**	0.5	0.5	**1**	3.25	0.75	**4**	0	0	**0**
	21	5	0	1	0	**6**	0.5	0	**0.5**	3.5	0	**3.5**	1	0	**1**
	22	2.25	2.5	2	0	**6.75**	0.5	0	**0.5**	4.25	0.5	**4.75**	0	0	**0**
	23	1.5	0.75	3	0.75	**6**	0.5	0.5	**0.5**	3.25	0.75	**4**	0	0	**0**
	24	1.75	1.75	3	0	**6.5**	0.25	0.25	**0.5**	2.25	2.75	**5**	0	0	**0**
	25	2.25	3	1.25	0	**6.5**	1	0	**1**	2.75	2	**4.75**	0	0	**0**
	26	3.5	0.5	2.5	0	**6.25**	0.5	0	**0.5**	3.75	0.25	**4**	0	0	**0**
	27	1.25	0.75	3.75	0.25	**6**	0.75	0	**0.75**	4.75	0.25	**5**	0	0	**0**

Continued…

School	Student	at home (hours)				Total	transit (hours)		Total			Total	Others		Total
		Living room	Kitchen/dinner room	Bedroom	Others (home)		for schooling	for others		classroom	outside classroom		indoor	Outdoor	
SU2	28	2.5	1.75	1.25	1.25	**6.75**	0.5	0	**0.5**	3	1.75	**4.75**	0	0	**0**
	29	1.75	1.25	1.25	2.25	**6.5**	0.25	0.25	**0.5**	3.75	0.25	**4**	0	1	**1**
	30	1.5	3	1.5	1	**7**	0.25	0.5	**0.75**	2.5	1.75	**4.25**	0	3.75	**0**
	31	2.25	0.75	2	2.25	**7.25**	0.75	0	**0.75**	4	0.5	**4.5**	0	0	**0**
	32	1	0.5	3.5	1	**6**	0.25	0.25	**0.5**	4	0.25	**4.25**	1.25	0	**0**
	33	0	1.75	3.75	1.5	**7**	0.25	0	**0.25**	4	0.75	**4.75**	0	0	**0**
	34	2	2	0.75	0.5	**5.25**	0.25	0	**0.25**	3.5	0	**3.5**	0	0	**1**
